# Are Nanoparticles a Threat to Mycorrhizal and Rhizobial Symbioses? A Critical Review

**DOI:** 10.3389/fmicb.2019.01660

**Published:** 2019-07-24

**Authors:** Hui Tian, Melanie Kah, Khalil Kariman

**Affiliations:** ^1^Key Laboratory of Plant Nutrition and Agri-environment in Northwest China, Ministry of Agriculture, College of Natural Resources and Environment, Northwest A&F University, Yangling, China; ^2^School of Environment, The University of Auckland, Auckland, New Zealand; ^3^School of Agriculture and Environment, The University of Western Australia, Crawley, WA, Australia

**Keywords:** nanoparticles, soil, mycorrhiza, rhizobia, colonization, nodule, toxicity

## Abstract

Soil microorganisms can be exposed to, and affected by, nanoparticles (NPs) that are either purposely released into the environment (e.g., nanoagrochemicals and NP-containing amendments) or reach soil as nanomaterial contaminants. It is crucial to evaluate the potential impact of NPs on key plant-microbe symbioses such as mycorrhizas and rhizobia, which are vital for health, functioning and sustainability of both natural and agricultural ecosystems. Our critical review of the literature indicates that NPs may have neutral, negative, or positive effects on development of mycorrhizal and rhizobial symbioses. The net effect of NPs on mycorrhizal development is driven by various factors including NPs type, speciation, size, concentration, fungal species, and soil physicochemical properties. As expected for potentially toxic substances, NPs concentration was found to be the most critical factor determining the toxicity of NPs against mycorrhizas, as even less toxic NPs such as ZnO NPs can be inhibitory at high concentrations, and highly toxic NPs such as Ag NPs can be stimulatory at low concentrations. Likewise, rhizobia show differential responses to NPs depending on the NPs concentration and the properties of NPs, rhizobia, and growth substrate, however, most rhizobial studies have been conducted in soil-less media, and the documented effects cannot be simply interpreted within soil systems in which complex interactions occur. Overall, most studies indicating adverse effects of NPs on mycorrhizas and rhizobia have been performed using either unrealistically high NP concentrations that are unlikely to occur in soil, or simple soil-less media (e.g., hydroponic cultures) that provide limited information about the processes occurring in the real environment/agrosystems. To safeguard these ecologically paramount associations, along with other ecotoxicological considerations, large-scale application of NPs in farming systems should be preceded by long-term field trials and requires an appropriate application rate and comprehensive (preferably case-specific) assessment of the context parameters i.e., the properties of NPs, microbial symbionts, and soil. Directions and priorities for future research are proposed based on the gaps and experimental restrictions identified.

## Introduction

Nanotechnology is starting to affect virtually every single aspect of our life, nearly 60 years after Richard Feynman articulated the concept of nanotechnology in his seminal 1959 lecture entitled “There’s plenty of room at the bottom”(Feynman, 1960). Nanotechnology has the potential to truly revolutionize agricultural systems by offering diverse applications such as nanofertilizers; nanopesticides; NP-based plant growth stimulators such as TiO_2_ NPs, SiO_2_ NPs, and carbon nanotubes; nanocarriers for targeted delivery and controlled release of agrochemicals; nanosensors for detecting early symptoms of biotic/abiotic stresses in plants; improving soil quality; and instrumental implications in efficient genetic manipulation techniques (Pérez-de-Luque and Hermosín, 2013; Fraceto et al., 2016). Crop growth promotion and protection against stresses are the core benefits of nanoagrochemicals, which can also make significant contributions to precision agriculture by reducing the quantity of agrochemicals applied to crops, wastage, and environmental pollution (Fraceto et al., 2016), though the environmental impact might not necessarily be mitigated (Kah et al., 2018).

Large-scale and versatile applications of NPs have inevitably led to their increasing presence in the environment and consequent risks (Nowack, 2009), which are of utmost significance from health and environmental perspectives. Nanoparticles entry into soil can occur through application of nanoagrochemicals, NP-containing amendments (e.g., biosolids, sludge and manure), and contamination by industrial wastes, plant litter, animal feces, carcasses, exuviae, and atmospheric deposition (Nowack et al., 2012; Qiu, 2012; McKee and Filser, 2016; Bundschuh et al., 2018). Hence, development of NPs for specific applications needs to be accompanied by ecotoxicological and biosafety studies on the interactions between NPs and biological components of the potentially affected environments. In the context of global agriculture, the fundamental question is “whether nanotechnology revolution can help us overcome the major challenge that agriculture is facing today i.e., feeding the world’s booming population without compromising soil health and sustainability?” To partly address this challenge, we critically assess the research literature dealing with the effects of different NPs on key plant-microbe symbioses namely mycorrhizas and rhizobia, which are of major ecological significance and are vital for functionality, productivity, and resilience of terrestrial ecosystems.

**FIGURE 1:**
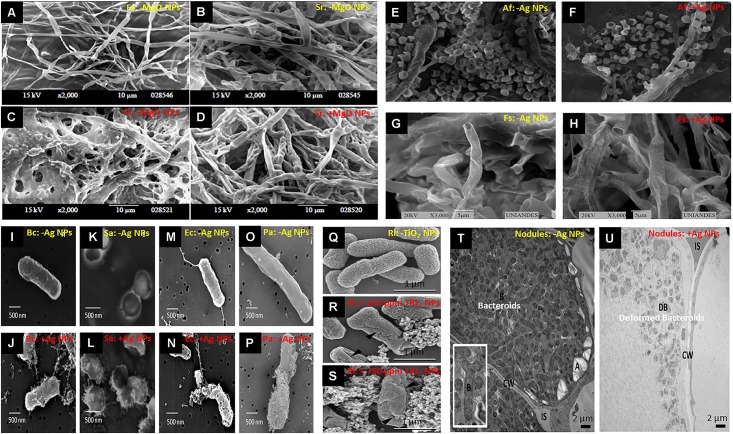
Deformation and damage in fungal hyphae and bacterial cells upon exposure to nanoparticles. **(A–D)** Scanning electron microscopy (SEM) of hyphae of *Fusarium solani* and *Sclerotium rolfsii*, untreated **(A,B)** or treated **(C,D)** with 100 ppm of magnesium oxide nanoparticles (MgO NPs), respectively. Some hyphae disintegrated and unusual bulges formed on the surface of fungal hyphae [adapted from El-Argawy et al. (2017)]. **(E,F)** SEM images of *Aspergillus flavus* before and after treatment with 50 ppm of silver nanoparticles (Ag NPs), showing dwindling of conidia; **(G,H)** SEM images of *Fusarium solani* before and after treatment with 50 ppm of Ag NPs, showing hyphal deformation [adapted from Villamizar-Gallardo et al. (2016)]. **(I–P)** SEM images of different bacteria, untreated or treated with bactericidal concentrations of Ag NPs: *Bacillus cereus* **(I,J)**, *Staphylococcus aureus* **(K,L)**, *Escherichia coli* **(M,N),** and *Pseudomonas aeruginosa* **(O,P)**, indicating membrane damage [adapted from Gopinath et al. (2012)]. **(Q–S)** Cell surface structure of *Rhizobium leguminosarum* bv. *viciae* 3841 shown by SEM micrograph in untreated control, and following exposure to 250 or 750 mg L^–1^ of titanium oxide (TiO_2_) NPs, respectively. (▲) indicates cracks and wrinkles caused by TiO_2_ NPs [adapted from Fan et al. (2014)]. **(T,U)** Transmission electron micrographs (TEM) of the infected zone of untreated and Ag NP-treated nodules, respectively, indicating digestion of peribacteroid membrane and deformed bacteroids (DB) in nodules of treated plants [adapted from Abd-Alla et al. (2016)].

**FIGURE 2:**
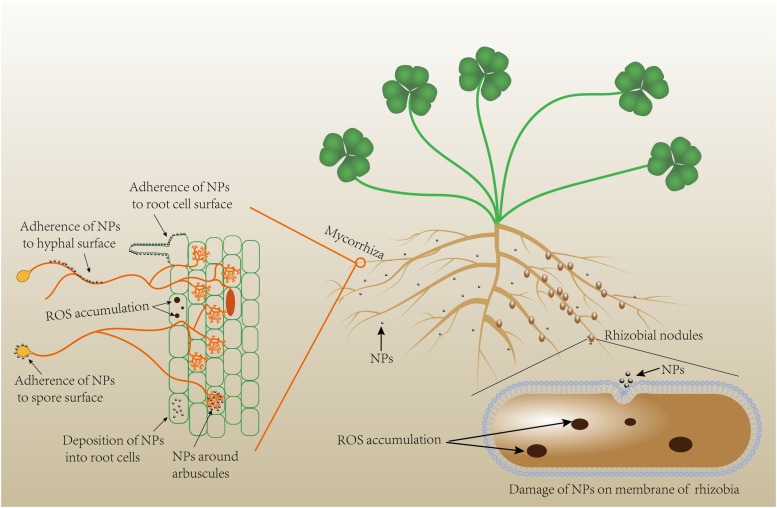
A theoretical demonstration of NPs exposure to roots, and root microbial symbionts. ROS, reactive oxygen species.

Nanoparticles are atomic or molecular aggregates of which a single unit measures from 1 to <1000 nm (in at least one dimension), but they usually are sized between 1 and 100 nm (Buzea et al., 2007). Due to their ultrafine size, NPs possess unique properties that are normally different from their respective bulk counterparts, which include large specific surface area (very high surface-to-volume ratio), high surface energy, and quantum confinement (Auffan et al., 2009). Nanoagrochemicals, including nanofertilizers, nanopesticides, nanocarriers, and NP-based growth stimulators, that are potentially more efficient and less contaminant than their conventional analogs have been synthesized and researched worldwide (Fraceto et al., 2016; Dimkpa and Bindraban, 2018; Kah et al., 2018). Positive, neutral, or negative responses have been documented in plants exposed to NPs, driven by the NPs type, size, dose, application methods, the target plant species, and experimental conditions (Hatami et al., 2016; Kah et al., 2018). Antimicrobial properties of certain NPs (such as Ag NPs, TiO_2_ NPs, and ZnO NPs) against bacteria and fungi are well acknowledged. Nanoparticles can deform and damage fungal hyphae and bacterial cells (Figure 1). Hence, NPs presence in soil might lead to reduced diversity and function of soil microorganisms (Ge et al., 2011; Kumar et al., 2011; Navale et al., 2015; Asadishad et al., 2018). However, there are also studies indicating limited (Tong et al., 2007; Johansen et al., 2008), or even positive effects of NPs on soil microbial communities and functioning (He et al., 2011; Karunakaran et al., 2013), proposing that NP-microbe interactions are context-driven. The root-microbe symbioses reside in the rhizosphere where factors such as interactions among the soil biota, resource complexity and availability, and biophysical heterogeneities may influence the magnitude of the NP effects on both free-living and root-colonizing microbes (Saleem et al., 2015; Raffi and Husen, 2019). Figure 2 illustrates the NPs exposure to roots and root microbial symbionts.

Mycorrhizal and rhizobial symbioses, arguably the most important symbioses on earth, are indispensable functional guilds of terrestrial ecosystems and continue to play a vital role in soil nutrient cycling, mineralization of organic matter, shaping plant and microbial communities, and ultimately safeguarding the functionality and resilience of the ecosystems (Finlay, 2008; van der Heijden et al., 2008). To date, a multitude of beneficial root-fungal symbioses have been described based on the properties of interface structures and the plant-fungus species involved (Smith and Read, 2008; Kariman et al., 2018). In the nanotechnology context, however, most research has primarily focused on the ubiquitous mycorrhiza i.e., arbuscular mycorrhiza, with a few studies partially dealing with other associations. Approximately, 80% of all vascular plant species establish arbuscular mycorrhizal (AM) symbiosis including bryophytes, pteridophytes, gymnosperms and angiosperms (Brundrett and Tedersoo, 2018) with fungi belonging to Glomeromycotina, a subphylum within Mucoromycota (Spatafora et al., 2016). Assorted benefits of AM symbiosis for host plants include access to the soil nutrients that are otherwise unavailable to roots (in particular, those with low mobility such as P and Zn), improved plant growth, and resistance against biotic/abiotic stresses such as pathogens, drought, salinity and heavy metals toxicity (Hildebrandt et al., 2007; Smith and Read, 2008; Miransari, 2010). Plant growth and reproduction can be gravely restricted by shortage of biologically active forms of N in soil. Certain plants have developed an efficient strategy to cope with N deficiency in soil. Plants from *Fabaceae* family enter into a symbiotic relationship with soil bacteria (including *Rhizobium*, *Bradyrhizobium*, *Sinorhizobium*, and *Burkholderia)*, which converts the atmospheric N_2_ to ammonia, referred to as rhizobial symbiosis (Oldroyd et al., 2011). Rhizobial symbioses make significant contributions to N nutrition of *Fabaceae* (the third largest plant family), legume crops and non-legume crops grown in rotation, and tolerance against environmental stresses such as heavy metals, organic pollutants and acidity (Crews and Peoples, 2004; Teng et al., 2015; Ramirez et al., 2018). At the symbiotic interface, roots form nodules to accommodate N_2_-fixing rhizobia. To initiate the legume-rhizobia symbiosis, nodule organogenesis and bacterial infection must be coordinated spatially and temporally, and need to be preceded by recognition of the rhizobial signaling molecule (a decorated lipochitooligosaccharide) called NOD Factor (Oldroyd and Downie, 2008). Rhizobial symbioses are sustainable N providers for legumes, and non-legume crops involved in intercropping systems or sequential rotations, and it is crucial to monitor their performance when exposed to NP-containing agrochemicals, amendments, or pollutants.

**FIGURE 3:**
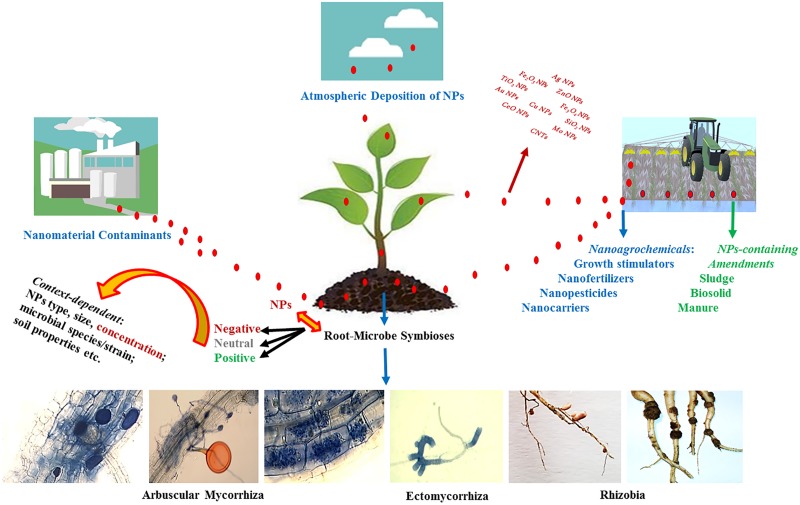
A schematic diagram showing the routes by which mycorrhizas and rhizobia may encounter nanoparticles (NPs) in soil, and the possible context-dependent consequences.

The net effect of NPs on development of mycorrhizal and rhizobial symbioses appear to be highly context-driven. While several studies indicated detrimental effects of NPs on mycorrhizal and rhizobial associations (Huang et al., 2014; Abd-Alla et al., 2016; Jing et al., 2016; Wang et al., 2016; Noori et al., 2017), there are also reports on the stimulatory impact of NPs (Feng et al., 2013; Chen et al., 2017), suggesting potentials to be harnessed for these beneficial root-microbe symbioses. Figure 3 displays routes by which mycorrhizal fungi and rhizobia may encounter NPs in soil, and the possible context-dependent consequences. In this review, we critically evaluate the existing literature dealing with the effects of NPs on development of mycorrhizal and rhizobial symbioses, identify the potential drivers of the interactions, and direct the future research toward minimizing the risks and harnessing the potential opportunities.

## Effects of Nanoparticles on Mycorrhizal/Rhizobial Symbioses, and the Influencing Factors

While there is great variability in the responses of plant associations with AM fungi and rhizobia to NPs, there are also clear patterns in what drives this variability. Our detailed assessment of the experimental conditions suggests that the net outcome of NPs on mycorrhizal colonization or nodule development is highly context-dependent and vary according to the NP properties, concentration, fungal/bacterial species, and characteristics of the interaction matrix (mostly soil) in which mycorrhizas/rhizobia live and interact with plant roots. There are very few studies on potential impact of NPs on the functioning of these symbiotic associations, so here we focus on the structure (i.e., the proportion of root length colonized by mycorrhizal fungi, or nodule formation on roots). The existing evidence, however, suggests that a balanced focus on both structural and functional traits is required in studies dealing with toxicology of NPs against these beneficial root-microbe symbioses. For instance, exposure of soybean (*Glycine max*) plants to a range of CeO NP concentrations (10−100 g kg^–1^ soil) did not affect nodulation, however, N fixation was shut down at medium and high concentrations (50 and 100 g kg^–1^ soil) (Priester et al., 2012). This highlights the fact that rhizobial (and perhaps mycorrhizal) development might appear to be unaffected by NPs, but they might not necessarily be functional. Below, we explore the potential factors driving the effects of NPs on mycorrhizal/rhizobial development by stating the supporting evidence. The reviewed studies have been conducted in controlled environments (e.g., glasshouse, lab, etc.), hence, we also highlight the experimental considerations that would direct future research closer to real-world scenarios.

## Nanoparticles Properties

Physicochemical properties of NPs (e.g., type, speciation, and size) strongly affect their impact on root mycorrhizal fungal colonization or nodulation. For example, Ag NPs seem to exhibit greater toxicity against mycorrhizas (Abd-Alla et al., 2016; Noori et al., 2017) compared with ZnO NPs (Li et al., 2015; Jing et al., 2016; Wang et al., 2016), considering their negative impact on root colonization at approximately 5−600 times lower concentrations in soil. Exposure to Fe_2_O_3_ NPs at 6 g L^–1^ did not affect nodulation in a symbiosis between pea and *Rhizobium leguminosarum.* 35 days after treatment, whereas the similar concentration and exposure time for ZnO NPs and TiO_2_ NPs led to negative effects on nodule development (Sarabia-Castillo and Fernández-Luqueño, 2016). CeO NPs application at 50 g kg^–1^ did not affect nodulation in soybean-*Bradyrhizobium japonicum* symbiosis, while the same concentration of ZnO NPs improved nodulation (Priester et al., 2012; Table 1). Bioavailability and potential effects of NPs on mycorrhizas/rhizobia might differ according to the NPs speciation and coating. For instance, PVP-Ag NPs (functionalized Ag NPs prepared via coating Ag NPs with polyvinyl pyrrolidone) were shown to reduce AM fungal colonization in tomato roots, while no significant effect was recorded for Ag_2_S NPs (silver sulfide NPs) at the same application rate of 100 mg kg^–1^ soil (Judy et al., 2015). Burke et al. (2015) used amine- and carboxylic acid-functionalized Fe_3_O_4_ NPs (carrying positive and negative surface charges, respectively) to investigate NP-rhizobia interactions, and observed that positively charged Fe_3_O_4_ NPs enhanced nodulation in soybean, compared to the negatively charged Fe_3_O_4_ NPs. This suggests that surface modification and coating of the NPs prior to exposure could affect the NPs toxicity against mycorrhizas/rhizobia, and therefore NP physicochemical properties can be modified to achieve a favorable outcome or avoid unwanted consequences in NP-mycorrhiza/rhizobia interactions.

**TABLE 1:** Effects of different nanoparticles on development of rhizobial symbioses.

**Symbiotic partners**	**Nanoparticle**	**Base element**	**Application dose mg kg^–1^/L^–1^**	**Media**	**Effect on nodulation**	**References**
Faba bean-*Rhizobium leguminosarum*	Ag	Silver	0.8	Sandy soil-loam mixture	Negative	Abd-Alla et al., 2016
Alfalfa-*Sinorhizobium meliloti*	Ag	Silver	5, 10	Jensen N free agar medium	Negative	Mohaddam et al., 2017
Soybean-*Bradyrhizobium japonicum*	CeO	Cerium	10,000 50,000 100,000	Farm soil	Neutral Neutral Neutral	Priester et al., 2012
Bean-*Rhizobium leguminosarum*	Cu (OH)_2_ (Kocide)	Copper	1.7 3.4 6.8	Sandy clay loam soil	Neutral Negative Negative	Baijukya and Semu, 1998
Soybean-*Bradyrhizobium japonicum*	Fe_3_O_4_	Iron	20−100	Nutrient solution	Positive	Ghalamboran, 2011
Soybean-unspecified rhizobia	Fe_3_O_4_	Iron	100, 200	Potting mix-sand-field soil mixture	Neutral	Burke et al., 2015
Pea-*Rhizobium leguminosarum*	Fe_2_O_3_	Iron	3,000^a^ 6,000^a^	Vermiculite	20 days: Negative 35 days: Positive 20 days: Neutral 35 days: Neutral	Sarabia-Castillo and Fernández-Luqueño, 2016
Chickpea-*Bradyrhizobium japonicum*	Mo	Molybdenum	≤8	Sandy loam soil	Positive	Taran et al., 2014
Pea-*Rhizobium leguminosarum*	TiO_2_	Titanium	100−750	Nutrient solution	Negative	Fan et al., 2014
Soybean-unidentified rhizobia	TiO_2_	Titanium	100−200	Potting mix-sand-field soil mixture	Neutral	Burke et al., 2015
Pea-*Rhizobium leguminosarum*	TiO_2_	Titanium	3,000^a^ 6,000^a^	Vermiculite	20 days: Negative 35 days: Neutral 20 days: Negative 35 days: Negative	Sarabia-Castillo and Fernández-Luqueño, 2016
Soybean-*Bradyrhizobium japonicum*	ZnO	Zinc	5,000 10,000 50,000	Farm soil	Neutral Neutral Positive	Priester et al., 2012
Pea- *Rhizobium leguminosarum*	ZnO	Zinc	200−800	Nutrient solution	Negative	Huang et al., 2014
Alfalfa-*Sinorhizobium meliloti*	ZnO	Zinc	50−100	Jensen N free agar medium	Negative	Mohaddam et al., 2017
Pea-*Rhizobium leguminosarum*	ZnO	Zinc	3,000^a^ 6,000^a^	Vermiculite	20 days: Negative 35 days: Negative 20 days: Negative 35 days: Negative	Sarabia-Castillo and Fernández-Luqueño, 2016
Barrel medic-*Sinorhizobium meliloti*	ZnO+TiO_2_+Ag	Zinc, Titanium, Silver	∼5,000: Ag ∼50,000: ZnO ∼50,000:TiO_2_	Soil amended with biosolids	Positive	Chen et al., 2017

*^*a*^Seedlings were grown in vermiculite medium and watered with NP aqueous solutions of the respective concentrations (mg L^–1^).*

Size of NPs was also found to influence NP-mycorrhiza interactions. Exposure to 2 nm-Ag NPs negatively affected root colonization in tomato, whereas a nil impact was observed for the larger Ag NPs of 15 nm at the same concentration of 12 mg kg^–1^ soil (Noori et al., 2017). Furthermore, TiO_2_ NP types differing in their size and crystalline structure were shown to move differently in soil, and the soil spiked with E171-TiO_2_ NPs (28 nm on average, consists of both anatase and rutile phases) had substantially elevated concentrations of Ti in the microcosm leachates compared with P25-TiO_2_ NPs (91 nm on average, consists of only anatase) (Klingenfuss, 2014), suggesting that NPs size and structure can potentially affect their bioavailability for soil microbes such as mycorrhizal fungi. Our knowledge is gravely limited on how the size of NPs might affect NP-rhizobia interactions, which warrants investigation. Chemical transformation of NPs can also possibly affect rhizobia, as partially or fully transformed NPs might possess different toxicity potential compared to the corresponding pristine materials (Liu et al., 2012; Reinsch et al., 2012).

## Nanoparticles Concentration

Concentration of NPs in the soil is a critical factor driving the NP-mycorrhizas/ rhizobia interactions. Root AM fungal colonization was shown to be decreased (Li et al., 2015; Jing et al., 2016) or unaffected (Watts-Williams et al., 2014; Wang et al., 2016) following exposure to ZnO NPs. Lack of the toxic effects on AM fungal colonization in tomato (Watts-Williams et al., 2014) and maize (Wang et al., 2016) plants was probably due to low concentrations of ZnO NPs (25 and 400 mg kg^–1^ soil, respectively), whereas higher concentrations (500 to 3200 mg kg^–1^ soil) led to reduced colonization in maize (Li et al., 2015; Jing et al., 2016; Wang et al., 2016). While a low Ag NPs concentration (0.01 mg kg^–1^ soil) had no impact on AM fungal colonization in white clover (*Trifolium repens*), higher concentrations (0.1 and 1 mg kg^–1^ soil) significantly enhanced the extent of root colonization (Feng et al., 2013). A low concentration of FeO NPs (0.032 mg kg^–1^ soil) stimulated the AM fungal colonization in white clover while nil impact was observed at a 100-fold higher concentration (3.2 mg kg^–1^ soil).

Similar to the responses observed for mycorrhizal colonization, NPs concentration seems to play a role in rhizobia-NP interactions (Table 1), though there is limited experimental evidence. Increasing ZnO NPs concentration from 5 to 50 g kg^–1^ was accompanied by a shift from neutral to positive effects on nodulation in soybean plants inoculated with *Bradyrhizobium japonicum* (Priester et al., 2012). Nodulation and N_2_ fixation were not affected upon exposure to Kocide, a Cu-based fungicide containing a significant proportion of Cu NPs (Adeleye et al., 2014) at the recommended rate (1.7 mg kg^–1^) while a reduced nodulation was observed at higher application rates of 3.4 and 6.8 mg kg^–1^ (Baijukya and Semu, 1998). In negative NP-rhizobia interactions, the lack of a negative correlation between NPs concentration and nodule development (Table 1; Fan et al., 2014; Sarabia-Castillo and Fernández-Luqueño, 2016; Mohaddam et al., 2017) could possibly be because the employed concentration range was beyond the toxicity threshold of NPs under the respective experimental conditions (soil-less media) i.e., all the concentrations led to a negative impact on rhizobial symbioses.

## Fungal/Bacterial Species

Different microbial taxa might exhibit various responses to NPs. Documented evidence indicates different mycorrhizal fungal taxa may exhibit various responses to NPs. The AM fungus *Glomus caledonium* was found to be more tolerant than *G. versiforme* against ZnO NPs toxicity based on extent of the negative impact of ZnO NPs on root colonization (Wang et al., 2016), which was attributed to higher tolerance of *G. caledonium* to heavy metals such as Zn, Cu, Pb, and Cd (Wang et al., 2007). Furthermore, although a DNA sequence analysis revealed five ectomycorrhizal (ECM)-forming genera on untreated roots of Bishop pine (*Pinus muricata*) including *Laccaria*, *Thelephora*, *Rhizopogon*, *Tomentella*, and *Tuber*, only *Laccaria* was found on roots of plants grown in soil spiked with 350 mg Ag NPs kg^–1^ (Sweet and Singleton, 2015).

There is currently not enough evidence regarding the sensitivity of different rhizobial species/strains against NPs, however, comparative studies between rhizobia and other bacteria suggest that differential responses might occur. For instance, the antibacterial action of ZnO NPs was demonstrated to be species-dependent as they exhibited bacteriostatic (preventing the growth of bacteria) effect on *Pseudomonas putida* (Gajjar et al., 2009), whereas the impact on the N_2_-fixing bacterium *Sinorhizobium meliloti* was of bactericidal (killing the bacteria) nature (Bandyopadhyay et al., 2012). Different strains of *Escherichia coli* were shown to be extremely sensitive or resistant to Ag NPs (Ashraf et al., 2014).

## Substrate Properties

Substrate/soil physicochemical properties such as pH, ionic strength, clay and organic matter contents can influence the NPs mobility, dissolution, release of ions, agglomeration, aggregation, and potential effects on soil microorganisms (Klaine et al., 2008; Tourinho et al., 2012). However, scant attention has been paid to the possible role of soil properties in NP-mycorrhiza interactions. TiO_2_ NPs application (8 mg kg^–1^ soil) was shown to negatively affect AM fungal colonization in rice (Priyanka et al., 2017), which can be attributed to many factors such as binding of TiO_2_ NPs to roots (Priyanka et al., 2017), increases in root internal Ti concentration as Ti is highly toxic for root growth (Burke et al., 2014), or possibly the soil properties such as the soil cation exchange capacity (CEC) that can influence the Ti bioavailability for plants and microbes. The AM fungal colonization showed a recovering trend after 90 days of exposure to TiO_2_ compared to that of day 30, and 60 (Priyanka et al., 2017) that was ascribed to high mobility of TiO_2_ in soil (i.e., the sandy soil led to a loss of TiO_2_ via leaching) (Mura et al., 2013) and/or formation of new roots in the absence of a high concentration of TiO_2_ NPs.

Negative (Abd-Alla et al., 2016), neutral (Judy et al., 2015), or positive (Feng et al., 2013) effects on AM fungal colonization of plants exposed to a similar level (∼ 1 mg kg^–1^ soil) of Ag NPs, could possibly be related to the totally different soil types used for these studies in which NPs bioavailability and fate might substantially differ (Klaine et al., 2008). AM fungal communities in the rhizosphere of soybean plants were shown to be altered following exposure to TiO_2_ NPs (Burke et al., 2014). However, no change was reported for the AM fungal community composition inside the soybean roots (Burke et al., 2015). The latter was ascribed to low Ti concentration in the root (5−10 μ⋅Ti⋅g^–1^ root tissue) compared to soil (200−400 μg⋅Ti⋅g^–1^), presumably due to the high CEC of the soil used for the study. Mycorrhizal communities within the roots were, therefore protected against the TiO_2_ NPs toxicity, which was presumably influenced by the soil CEC. Within the soil (or growth medium), NPs can be chemically transformed and form coronae that could potentially affect NP kinetics and behavior (Lowry et al., 2012). Li et al. (2017) demonstrated that in paddy soils spiked with PVP-Ag NPs, the presence of soil organic matter enhanced Ag retention in the soil solids and decreased the dissolved Ag levels, whereas high redox potential led to reduced Ag sulfidation and increased the release of dissolved Ag. Accordingly, the natural chemical transformation of NPs in soil and possible impacts on mycorrhizas need to be considered.

Soil nutrient status may also play a role in NP-mycorrhiza interactions. While ZnO NPs were toxic to AM fungal colonization (by *Funneliformis mosseae*) in maize at 500 mg kg^–1^ soil, addition of the bulk counterpart (ZnSO_4_, 500 mg kg^–1^ soil) led to growth promoting and protective effects of the AM symbiosis against ZnO NPs toxicity (Li et al., 2015). These examples signify a highly context-dependent impact of NPs on mycorrhizas, and also suggest that favorable outcomes can be achieved by manipulation of the influencing factors, where applicable.

Although the extent of root colonization is a universally recognized index for mycorrhizal development, it does not necessarily reflect the actual mycorrhizal functioning and mycorrhiza-mediated benefits for host plants such as nutrient gain and tolerance against environmental stresses (Jakobsen, 1995; Smith et al., 2004, 2009; Kariman et al., 2014a,b, 2018). To better understand the impact of NPs on mycorrhizas, along with the quantitative measurement of mycorrhizal development (i.e., root colonization), the mycorrhizal benefits for NP-treated plants need to be experimentally investigated.

Likewise, the properties of substrate/growth medium can have a dramatic effect on NP-rhizobia interactions. As summarized in Table 1, many studies on NP-rhizobia interactions have been conducted in soil-less media. Although insight into nodulation responses to NPs can be gained from soil-less studies, such piecemeal evidence is inadequate to infer how NPs might affect development and functioning of legume-rhizobia symbioses in complex soil environments in which NPs bioavailability and impact on microbial communities could vary dramatically (Tong et al., 2007; Ge et al., 2011; Abd-Alla et al., 2016). For instance, soil application of ZnO NPs at 50 mg kg^–1^ resulted in a positive impact on nodule development in soybean plants associated with *Bradyrhizobium japonicum* (Priester et al., 2012), whereas comparably lower concentrations of ZnO NPs had severe adverse effects on nodule development in plants grown in soil-less media such as vermiculite (Sarabia-Castillo and Fernández-Luqueño, 2016), hydroponic cultures (Huang et al., 2014), and Jensen N free agar medium (Mohaddam et al., 2017). Most studies indicating negative effects of NPs on rhizobial development have been carried out in soil-less media in which the supplemental NPs are highly available (with enhanced NPs dissolution kinetics compared to soil) for both host plants and rhizobia (Fan et al., 2014; Huang et al., 2014; Sarabia-Castillo and Fernández-Luqueño, 2016; Mohaddam et al., 2017). Adverse effects on soil microbial community composition (more evidently on Rhizobials) were detected in a sandy loam soil spiked with CuO NPs, whereas limited effects were observed for a sandy clay loam soil, which contained a higher clay proportion (Abd-Alla et al., 2016). The decreased toxicity of NPs against soil microorganisms has been attributed to certain soil properties such as high clay content (Schlich and Hund-Rinke, 2015; Abd-Alla et al., 2016), alkaline pH (Shen et al., 2015), and low organic matter content, presumably due to the instability of the NP aggregates (Simonin et al., 2015).

## Mechanisms Underlying the Effects of Nanoparticles on Mycorrhizal/Rhizobial Development

Our mechanistic understanding of NP-mycorrhiza/rhizobial interactions is limited (Figure 2). Some studies have attributed the negative impact of NPs on mycorrhizal colonization to antifungal activities of NPs including the release of metal ions such as Zn^+2^ (Wang et al., 2016) and Ag (Yin et al., 2011; Sweet and Singleton, 2015), binding of NPs to roots (Seeger et al., 2009; Priyanka et al., 2017), and increases in root internal NPs concentration and root growth inhibition (Burke et al., 2014; Priyanka et al., 2017). The adverse effects of NPs on mycorrhizal colonization could be directly due to the antifungal properties of NPs that include: adherence of NPs to the cell surface and physical damage to cell wall and membrane, increasing the membrane permeability, blocking the water channels, and cell death due to penetration and deposition of NPs into cells (Wang et al., 2014); cutting fungal structures and cell walls due to the sharp edges of NPs (Xie et al., 2016); inhibiting spore germination by forming NP-spore aggregates through van der Waals forces (Wang et al., 2014); accumulation of reactive oxygen species (ROS) via impairing the ROS-scavenging defense systems such as the cycle and regulation of glutathione (Akhavan and Ghaderi, 2012; Liu et al., 2016); release of ions from metal-based NPs (Kanhed et al., 2014; Hao et al., 2017; Zarzuela et al., 2017); and photocatalytic activities of certain NPs such as TiO_2_ (De Filpo et al., 2013).

Mycorrhizal colonization may decline following exposure of host plants to environmental stresses such as drought (Huang et al., 2017) and salinity (Wang et al., 2018) caused by a reduction in plant growth and photosynthesis, which is accompanied by reduced C allocation to root fungal symbionts. Other than the direct antifungal activities of NPs mentioned above, the decline in root colonization of NP-treated plants could be related to the possible negative effects of NPs on plant growth and fitness, where NPs exhibit phytotoxicity (Abd-Alla et al., 2016; Noori et al., 2017). Taken together, the negative effects of NPs on mycorrhizal development could be due to their direct (antifungal) and indirect (phytotoxic) effects.

Mechanisms behind the positive NP-mycorrhiza interactions are barely explored, and warrant investigation. Ag NPs were found to stimulate AM fungal colonization (Feng et al., 2013). An increase in AM fungal colonization has also been observed in plants under heavy metals stress (Vogel-Mikus et al., 2006). The stimulatory effect of Ag NPs (and heavy metals) on mycorrhizal colonization could be ascribed to additional C allocation to mycorrhizal fungi in order to benefit from their protective effects (Kiers et al., 2011).

Decline in rhizobial nodule development of plants exposed to NPs might be a direct consequence of the antibacterial activities of NPs. The antibacterial modes of action of NPs could generally be an outcome of oxidative stress induction (Gurunathan et al., 2012), metal ion release (Nagy et al., 2011; Huang et al., 2014), or non-oxidative mechanisms such as cell membrane damage (Fan et al., 2014; Leung et al., 2014), all of which can occur simultaneously. Morphological changes and cell surface damage in *Rhizobium leguminosarum* bv. *viciae* 3841 exposed to ZnO NPs was ascribed to adhesion of NPs onto cell walls, release of Zn^2+^ ions, and possible generation of ROS (Huang et al., 2014). Scanning electron microscopy (SEM) of cell surface structure of *Rhizobium leguminosarum* bv. *viciae* 3841 (Rlv 3841) showed that exposure to TiO_2_ NPs caused cracks and damage in cell membrane (Figures 1R,S; Fan et al., 2014). Transmission electron micrographs (TEM) of the infected zone of Ag NP-treated nodules indicated the digestion of peribacteroid membrane and presence of deformed/disintegrated bacteroids in nodules (Figure 1U; Abd-Alla et al., 2016). The decline in *pea-Rhizobium leguminosarum* symbiosis following exposure to TiO_2_ NPs was accompanied by morphological changes in the outer membrane of the rhizobia, changes in the composition of the cell wall polysaccharides of nodules, and a stress possibly caused by generation of hydroxyl radicals that could attach onto the nodules cell wall (Fan et al., 2014). These examples clearly emphasize on the antibacterial activities of NPs as a key mechanism behind the declined rhizobial symbiosis of the NP-treated plants. Nevertheless, as discussed about the mycorrhiza-NP interactions, the impact of NPs on host plants might also influence development of the rhizobial symbiosis. Environmental stresses such as drought can substantially decrease nodulation and N_2_ fixation as a consequence of C shortage, oxygen limitation, or feedback regulation by nitrogen accumulation (Serraj et al., 1999). Inhibition or retardation of the rhizobial symbiosis in pea plants exposed to ZnO NPs was shown to be linked with reductions in root and shoot growth (Huang et al., 2014; Sarabia-Castillo and Fernández-Luqueño, 2016) i.e., reduced growth and C allocation to microbial symbionts.

Limited evidence is available about the mechanisms underlying the stimulatory effects of NPs on nodule formation and development, but there have been some speculations. For instance, the enhanced nodule development in soybean plants exposed to positively charged Fe_3_O_4_ NPs was attributed to supply of Fe (Burke et al., 2015), which is essential for N_2_ fixing bacteria (Brear et al., 2013). In addition, nodule factor (nodf) and genistein (a major root-secreted isoflavone that induces the expression of *Bradyrhizobium japonicum* nod YABC operon) production were shown to be up-regulated by Fe_3_O_4_ NPs, which were suggested to be linked with the improved nodulation observed in the symbiosis between soybean plants and *Bradyrhizobium japonicum* (Ghalamboran, 2011).

## Benefits of Mycorrhizal and Rhizobial Symbioses for Plants Exposed to Toxic Levels of Nanoparticles

Arbuscular mycorrhizal fungal colonization generally enhances the activities of antioxidant enzymes such as superoxide dismutase (SOD) under heavy metal stress, thus reducing the oxidative damage to biomolecules via scavenging the generated ROS (Azcon et al., 2009). Reduced bioavailability of heavy metals (accompanied by reduced phytotoxicity) in plants colonized by AM fungi have been attributed to the fungal capacity in increasing soil pH and producing glomalin-related soil proteins that can bind to metals (Wang et al., 2012).

Although few studies have specifically monitored the benefits of mycorrhizas for host plants under NP toxicity, several lines of evidence indicate their positive role. ZnO NPs were shown to be toxic to AM development in maize plants, however, the symbiosis could still alleviate the phytotoxic effects of ZnO NPs by decreasing Zn bioavailability (via hyphal sequestration) and accumulation in plant tissues, Zn translocation to shoots, and ROS generation, as well as improving mineral nutrition (Mg, in particular) and antioxidant capacity of host plants (Wang et al., 2016). Under elevated levels of Ag NPs (concentrations over 0.1 mg kg^–1^), the AM fungus *G. caledonium* ameliorated the NPs stress in white clover plants as compared with the uninoculated control (Feng et al., 2013). AM fungal colonization was almost completely inhibited in the presence of Ag NPs and Ag^+^ ions (at 100 mg kg^–1^ soil), while it was not affected by the Ag_2_S NPs treatment (Judy et al., 2015). The latter is a typical example of differential interaction of pristine vs. transformed NPs with mycorrhizas. The Ag_2_S NP-treated plants had higher root colonization but lower shoot Ag concentrations, proposing a mycorrhiza-mediated tolerance against Ag_2_S NPs via reduced Ag uptake relative to uninoculated plants. Likewise, mycorrhizal tomato plants exposed to 36 mg kg^–1^ of Ag NPs (2 nm) accumulated 14% less Ag in their shoot tissues compared to non-mycorrhizal plants (Noori et al., 2017), and the expression of potassium channel (KC), plasma membrane intrinsic protein (PIP), and a tonoplast membrane intrinsic protein (TIP) genes in mycorrhizal plants was lower than that of the non-mycorrhizal control. These findings demonstrated that mycorrhizal colonization can decrease Ag accumulation in Ag NP-exposed plants and moderate changes in expression level of membrane transport proteins that are possibly involved in Ag uptake. Judy et al. (2016) demonstrated that AM fungi did not play a significant role in the transfer of Au NPs to tomato plants. Au NPs accumulated at the rhizoplane of plants that developed a robust (about 35%) AM fungal colonization, suggesting a low toxicity of Au NPs for AM fungi and their possible protective effects on plants. Likewise, mycorrhizal tomato plants (76R) supplied with ZnO NPs were shown to accumulate lower Zn in their shoot compared to the non-mycorrhizal tomato mutant (rmc) (Watts-Williams et al., 2014). These examples suggest that mycorrhizas can possibly confer tolerance to host plants against NP toxicity via reduced NP uptake. Nevertheless, Whiteside et al. (2009) showed that AM fungi were actively involved in uptake and transfer of quantum dots (QDs) to roots of the annual bluegrass (*Poa annua*), which could be a specific response to QDs due to their very small size and unique properties (Al-Salim et al., 2011).

Other than conferring protective effects to host plants against NP toxicity, AM fungi were shown to alleviate the toxic effects of NPs on other soil biota. No significant changes in the relative abundance of Planctomycea, Sphingobacteria, Chloracidobacteria, Acidobacteria and Actinobacteria were found under a toxic concentration of Fe_3_O_4_ NPs in plants colonized by AM fungi, whereas these bacterial taxa were altered in non-mycorrhizal plants (Cao et al., 2016). Moreover, the relative abundance of *Nitrospira* (nitrite-oxidizing bacteria) and *Anaerolineae* (organic matter-decomposing bacteria) increased significantly by AM symbiosis compared to non-mycorrhizal plants under Fe_3_O_4_ NPs treatment, which can potentially contribute to N and C cycling in soil, respectively. Overall, the above-mentioned evidence suggests that mycorrhizal symbiosis has the capacity to protect host plants, beneficial microbes, and maintain soil function under NP toxicity. Mycorrhizas may also enhance the efficiency of nanofertilizers. Growth stimulation by the nanofertilizer Ca_3_(PO_4_)_2_ NPs in maize was shown to be improved by the AM fungus *G. mosseae* and/or the sebacinalean endophyte *Serendipita indica* (Rane et al., 2015).

There is no direct experimental evidence on whether rhizobial symbioses could aid plants to survive and thrive under toxic levels of NPs because most studies have considered untreated controls (i.e., with rhizobia, with or without NPs), but not uninoculated controls (i.e., without rhizobia, with or without NPs). However, rhizobia possess the biochemical and ecological capacity to decrease the risks associated with metals, metalloids, and organic pollutants in contaminated soils (Teng et al., 2015). For instance, the acidic-Al tolerant *Burkholderia fungorum VTr35* strain was shown to induce tolerance to soybean plants against acid-Al stress conditions (Ramirez et al., 2018). Furthermore, rhizobia were shown to confer tolerance to host plants against heavy metals and oxidative stress through production of hydrogen (H_2_), which is a by-product of the symbiotic N_2_ fixation process and possess novel bioactive properties (Cui et al., 2013; Jin et al., 2013).

## Do Nanoparticles Levels that are Toxic to Mycorrhizas and Rhizobia Occurring in Soil?

Manifold examples of negative effects of NPs on mycorrhizal and rhizobial symbioses were presented (Tables 1, 2) and discussed. Table 3 compares the mycorrhiza-toxic concentrations of NPs with plant-promoting concentrations of nanoagrochemicals (i.e., the potential soil application rate), along with NP concentrations reported from NP-polluted soils. It can be concluded that most of the adverse effects on mycorrhizas have been observed at NP concentrations exceeding those that can be realistically expected in agroecosystems and the natural environments. With respect to rhizobia, most studies dealing with negative rhizobia-NP interactions (Table 1) have been carried out in soil-less media in which NPs bioavailability and effects might substantially differ from soil systems. Hence, studies using more realistic NP concentrations and soils with different physicochemical properties (instead of vermiculite, agar-based media, or nutrient solutions) would be of paramount significance to further elucidate the inhibitory or stimulatory effects of NPs on mycorrhizas and rhizobia.

**TABLE 2:** Effects of different nanoparticles on development of mycorrhizal symbioses.

**Mycorrhizal partners**	**Nanoparticles**	**Base element**	**Application dose mg kg^–1^/L^–1^**	**Media**	**Effect on root colonization**	**References**
White clover-*Glomus caledonium*	Ag	Silver	0.01 0.1, 1	Sand-perlite mixture	Neutral Positive	Feng et al., 2013
Faba bean-*Glomus aggregatum*	Ag	Silver	0.8	Sandy soil-loam mixture	Negative	Abd-Alla et al., 2016
Tomato-AMF^*^	Ag_2_S PVP-Ag	Silver- sulfidized Silver- PVP coated	1, 100 10 100 1, 10	Sandy loam-sludge mixture	Neutral Negative Negative Neutral	Judy et al., 2015
Tomato-AMF	Ag-2 nm Ag-15 nm	Silver	12, 24, 36 12 24, 36	Soil	Negative Neutral Negative	Noori et al., 2017
Tomato-AMF	Au	Gold	25	Sandy soil	Neutral	Judy et al., 2016
Red clover-AMF	Carbon nanotubes	Carbon	3, 3000	Sandy loam soil	Neutral	Moll et al., 2016
Red clover-AMF	CeO	Cerium	860	Sandy loam soil	Neutral	Moll et al., 2016
Tomato- *Funneliformis mosseae*	Chitosan- silica nanocomposites	Chitosan- Silica	Concentration unspecified: used as nanocarrier	Cocopeat	Positive	Gatahi et al., 2016
Clover-*Glomus caledonium*	FeO	Iron	3.2 0.032	Sand-perlite mixture	Neutral Positive	Feng et al., 2013
Wheat-AMF	TiO_2_	Titanium	1, 100, 1000	Sand-soil mixture	Neutral	Klingenfuss, 2014
Soybean-AMF	TiO_2_	Titanium	100, 200	Potting mix-soil mixture	Neutral	Burke et al., 2014
Rice-AMF consortium	TiO_2_	Titanium	8, 16, 33	Sandy soil	Negative	Priyanka et al., 2017
Red clover-AMF	TiO_2_	Titanium	10, 100, 1000	Sandy loam soil	Neutral	Moll et al., 2016
Tomato-AMF	ZnO	Zinc	25	Sand-soil mixture	Neutral	Watts-Williams et al., 2014
Maize-*Funneliformis mosseae*	ZnO	Zinc	500	Soil	Negative	Li et al., 2015
Soybean-*Funneliformis mosseae*	ZnO	Zinc	=2000	Soil	Negative	Jing et al., 2016
Maize-*Glomus versiforme/caledonium*	ZnO	Zinc	400 800−3200	Loamy soil	Neutral Negative	Wang et al., 2016

^*^“AMF” denotes unspecified AM fungal species; the species identity was provided, where possible.

**TABLE 3:** Mycorrhiza-toxic concentrations of nanoparticles vs. the predicted soil nanoparticles concentrations and/or plant-promoting soil application rates of nanoagrochemicals.

**Nanopar- ticle**	**Mycorrhiza-toxic concentration**	**Predicted soil concentration**	**Soil application rate (plant promoting rate range)**
ZnO	500−3200 mg kg^–1a,b,c^	Agricultural soils: 0.008−0.35 μg kg^–1d^	10−500 mg kg^–1f,g,h^
		Undisturbed soils: 0.018–0.9 μg kg^–1d^	
		Unspecified soil types: Switzerland: 0.026−0.661 Δ μg kg^–1^ y^–1e^	
TiO_2_	Likely ≥1000 mg kg^–1i,j,k^ Though negative effects once reported at 8 mg kg^–1l^	Agricultural soils: 0.01−1.7 μg kg^–1d^ Undisturbed soils: 0.024−4.9 μg kg^–1d^	20−300 mg kg^–1m,n,s^ Up to 1000 mg kg^–1o^
		Unspecified soil types: 0.21−4.45 Δ μg kg^–1^ y^–1e^	
Ag	10−100 mg kg^–p,q^ 800 μg kg^–1r^	Agricultural soils: 6−21 ng kg^–1d^	NA
		Undisturbed soils: 13−61 ng kg^–1d^	
		Unspecified soil types: 6.6−58.7 Δ ng kg^–1^ y^–1e^	

Δ*ng or μg kg^−1^ y^−1^: increase of NPs concentration per year (base year 2008). NA: not applicable. The superscript letters within the table correspond to the following references a, Jing et al. (2016); b, Li et al. (2015); c, Wang et al. (2016); d, Gottschalk et al. (2015); e, Gottschalk et al. (2009); f, Liu et al. (2015); g, Priester et al. (2012); h, Singh and Kumar (2016); i, Burke et al. (2015); j, Klingenfuss (2014); k, Moll et al. (2016); l, Priyanka et al. (2017); m, Hanif et al. (2015); n, Rafique et al. (2014); o, Raliya et al. (2015); p, Judy et al. (2015); q, Noori et al. (2017); r, Abd-Alla et al. (2016); s, Singh and Lee (2016).*

However, environmentally relevant concentrations of NPs were also shown to strongly affect soil microbial communities, critical ecosystem services such as nutrient turnover, and greenhouse gas emissions (McKee and Filser, 2016). In general, application of any substance that is persistent and immobile in soil should be considered extremely cautiously. Nanoparticles are elements and thus do not breakdown in the environment. For instance, if NP concentrations are one order of magnitude apart, toxic levels may be reached in soil after 10 years of consecutive applications (once a year). Many NPs appear to be persistent and rather immobile in soil depending on the NPs and soil properties, and also incorporate into soil biota and plant tissues (Lin et al., 2010; McKee and Filser, 2016). Accordingly, the effects of repetitive applications of NPs on these key root-microbe symbioses need to be considered for nanoagrochemicals and NP-containing amendments (e.g., Ag NPs added through sludge application) via conducting spatial and temporal trials.

Historical use of Cu-based fungicides over decades has resulted in Cu accumulation in soils up to one order of magnitude higher concentration relative to the natural soils, which can cause adverse environmental impacts on soil fertility, organisms, and contaminate ground/surface water resources (Dumestre et al., 1999; Wightwick et al., 2010). Future research needs to address a fundamental question: “Could NPs accumulation in soil upon repetitive applications be an environmental concern similar to what that has been reported for Cu following long-term application of Cu-based fungicides?

## Conclusion

Our in-depth evaluation of the literature shows that NPs may have negative, neutral or even positive effects on development of mycorrhizal and rhizobial symbioses. Most studies indicating adverse effects of NPs on mycorrhizas and rhizobia seem to have been performed using either unrealistically high NP concentrations that might not normally occur in soil, and/or irrelevant growth medium (mostly in rhizobial studies). A few reports also exist about the stimulatory impact of NPs on mycorrhizal/rhizobial development, suggesting that NPs might also be purposely used to promote these ecologically paramount associations.

The net effects of NPs on mycorrhizal colonization depend on various factors including NPs type, speciation, size, concentration, fungal species/strain, and the physicochemical properties of the soil or substrate. However, as expected for many potentially toxic compounds, NPs concentration was found to be the most crucial factor determining NP toxicity against mycorrhizas, as even less toxic types (e.g., ZnO NPs) could become toxic at high concentrations, or highly toxic types (e.g., Ag NPs) could be beneficial at low concentrations. Likewise, rhizobial responses to NPs are highly context-driven and depend on the concentration and properties of NPs, rhizobia, and the growth substrate. However, use of inappropriate growth media (e.g., hydroponic cultures) coupled with lack of nano-specific quality assurance and appropriate controls prevent us from drawing firm conclusions. Lack of nano-specific quality assurance is indeed a crucial issue. Nanoparticles need to be characterized as close as possible to the conditions of exposure. Many studies lacked adequate characterization, which prevents the elucidation of the mechanisms involved and comparison of the results of different studies. Thorough understanding of the key drivers of NP-mycorrhizas/rhizobia interactions would help us manage the possible consequences toward protection and/or promotion of these beneficial associations.

It is crucial to exploit the promising potential of nanotechnology to improve global food security and diminish the environmental footprint of modern agriculture. To maintain ecosystem functioning and resilience, research needs to be intensified on interactions between NPs and these key root-microbe symbioses. We have identified the future research priorities and experimental considerations as follows:

• Experiments should be conducted under more realistic experimental conditions. For instance, future studies should consider using agronomically/environmentally relevant NP concentrations in soils representing a range of properties (rather than soil-less systems).
• Adequate controls should be considered, including treatments with or without mycorrhizal fungi/rhizobia and NPs, and comparisons with non-nano analogs and currently used agrochemicals.
• A broader range of endpoints representing both structural development and functionality of the symbioses in NP-treated plants should be considered in the future studies.
• To investigate the functionality of mycorrhizal/rhizobial associations exposed to NPs, factors linked to viability and functionality of the symbioses need to be explored including the germination of mycorrhizal fungal spores, hyphal growth and function, multiplication of bacteroids in rhizobial nodules, and nutritional symbiotic benefits.
• Transformation and accumulation of NPs in soil over time and their long-term effects on mycorrhizal fungi and rhizobia need to be monitored, particularly for NPs that are intentionally and potentially repeatedly applied.
• Testing aged/transformed NPs relative to pristine NPs, and hence, considering the appropriate environmental media (soil) and time scale that allow these transformation processes to take place.
• Greater attention should be directed toward NP-based agrochemicals such as nanofertilizers, nanopesticides, plant growth stimulators (such as TiO_2_ NPs, SiO_2_ NPs, and carbon nanotubes) and organic nanocarriers (e.g., chitosan, graphene oxide, polymers etc.), because their concentrations in soil are more likely to cross the mycorrhizas/rhizobia-toxic threshold rather than those NPs originating from environmental pollutants such as Ag NPs.
• Most studies have focused on AM symbiosis and a crucial knowledge gap exists about interactions between NPs and other mycorrhizal associations. Bearing in mind the unavoidable release of NPs in natural ecosystems, it is essential to explore the interactions between NPs and the other key mycorrhizal associations such as ECM and orchid mycorrhizas.
• Whether or not mycorrhizal fungal and rhizobial strains can become resistant against NPs following repeated applications over time is unknown and deserves further investigations.

## Author Contributions

HT conceptualized the manuscript. HT and KK wrote the manuscript. MK provided valuable input and insight on different components of the manuscript.

## Conflict of Interest Statement

The authors declare that the research was conducted in the absence of any commercial or financial relationships that could be construed as a potential conflict of interest.
